# Associations between Intra-Assessment Resting Metabolic Rate Variability and Health-Related Factors

**DOI:** 10.3390/metabo12121218

**Published:** 2022-12-04

**Authors:** Juan M. A. Alcantara, Francisco J. Osuna-Prieto, Abel Plaza-Florido

**Affiliations:** 1 PROFITH “PROmoting FITness and Health through Physical Activity” Research Group, Department of Physical and Sports Education, Faculty of Sport Sciences, Sport and Health University Research Institute (iMUDS), University of Granada, 18011 Granada, Spain; 2 Department of Health Sciences, Institute for Innovation & Sustainable Food Chain Development, Public University of Navarra, Campus Arrosadía, s/n., 31006 Pamplona, Spain; 3 Navarra Institute for Health Research, IdiSNA, 31008 Pamplona, Spain; 4 Department of Analytical Chemistry, University of Granada, 18071 Granada, Spain; 5 Pediatric Exercise and Genomics Research Center, Department of Pediatrics, School of Medicine, University of California at Irvine, Irvine, 92617 CA, USA

**Keywords:** metabolic cart, CCM Express, CPX Ultima CardiO2, indirect calorimetry, sexual dimorphism, cardiovascular diseases

## Abstract

In humans, the variation in resting metabolic rate (RMR) might be associated with health-related factors, as suggested by previous studies. This study explored whether the intra-assessment RMR variability (expressed as a coefficient of variation (CV; %)) is similar in men and women and if it is similarly associated with diverse health-related factors. The RMR of 107 young, and relatively healthy adults, was assessed using indirect calorimetry. Then, the CV for volumes of oxygen consumption (VO_2_) and carbon dioxide production (VCO_2_), respiratory exchange ratio (RER), and resting energy expenditure (REE) were computed as indicators of intra-assessment RMR variability. Body composition, cardiorespiratory fitness (peak VO_2_ uptake), circulating cardiometabolic risk factors, and heart rate and its variability (HR and HRV) were assessed. Men presented higher CVs for VO_2_, VCO_2_, and REE (all *p* ≤ 0.001) compared to women. Furthermore, in men, the intra-assessment RER variability was associated with vagal-related HRV parameters and with mean HR (standardized β = −0.36, −0.38, and 0.41, respectively; all *p* < 0.04). In contrast, no associations were observed in women. In conclusion, men exhibited higher variability (CVs for VO_2_, VCO_2_, and REE) compared to women. The CV for RER could be a potential marker of cardiometabolic risk in young men.

## 1. Introduction

It is well known that the energy cost of self-maintenance varies within species, within and between days, and between sexes [1,2,3,4]. This self-maintenance component of energy cost is commonly referred to as the basal or resting metabolic rate (RMR). Nevertheless, whether this is a “basal” or a “resting” assessment depends on the methodology followed in the experiment [5,6]. The RMR, which is widely defined as the minimum energy needed for maintaining body homeostasis and normal body functions (organ functions, thermoregulation, etc.), may account for 60–70% of the 24-h energy expenditure of sedentary individuals [7].

The RMR component can be assessed in a relatively easy manner using indirect calorimetry and metabolic carts [8], which are considered the reference tool for its assessment [5,6]. Thus, metabolic carts allow us to assess and study the RMR of individuals. The variation in RMR between species is undoubtedly and mostly explained by differences in body mass, although other factors may also influence these differences [1,2,3,9]. In mammals (e.g., humans), the variation in RMR appears to influence behavioral traits and fitness (e.g., peak volume of oxygen consumption [VO_2_] uptake), among others [10,11,12,13]. Importantly, the RMR seems to be a repeatable component over time, showing within- and between-day reproducibility of 3–8%—that reproducibility percentage range corresponds to humans’ RMR assessments using a metabolic cart system [9]. Nowadays, in humans, studying the differences between sexes, and, more concretely, the variability in energy expenditure (EE), is a matter of interest. In a study by Halsey et al. [14], they observed a greater men vs. women variability in total EE, activity EE (estimated as: 0.9 × total EE − basal EE, aiming to determine EE cost from physical and/or exercise), and basal EE components. Interestingly, even after comparing men and women of the same age, height, and fat and fat-free mass-characteristics that directly influence EE—men exhibited more variability than women [14].

Concerning RMR assessments using metabolic carts, the variability (expressed as the coefficient of variation (CV) in percentage) of the measured parameters, such as the VO_2_, the volume of carbon dioxide production (VCO_2_), and the respiratory exchange ratio (RER), have also been of interest. In fact, the CV for these parameters is widely employed as criteria for determining the “gas exchange stability” and, thus, as a cut-off point for selecting the RMR data [15]. Of note, the CV for RMR itself (i.e., the CV for resting EE) is not commonly used as a cut-off point for this data selection. A previous study by Irving et al. [16], conducted in healthy participants, showed that 12 individuals (16% of the sample) exhibited elevated intra-assessment variability (CV for VO_2_ and VCO_2_ > 10%) during the entire 45-min RMR assessment. Intriguingly, they observed that 17% of them presented extreme low and high body mass index (BMI) values (BMIs < 17.5 and > 48, respectively) [16]. Thus, they suggested that an extreme BMI could influence the intra-assessment RMR variability, i.e., the higher the BMI, the higher the CVs for VO_2_ and VCO_2_. In addition, Reeves et al. [17] observed that 55% out of a sample of 39 participants did not accomplish the gas exchange stability criteria, or, in other words, they presented an elevated intra-assessment variability. It is important to acknowledge that more than half of the study cohort were cancer patients, which may influence that intra-assessment variability to an unknown extent [17]. In this line, the more ill the participant, the greater the variability in the RMR [17,18,19,20,21,22,23], an observation supported by studies performed in different populations with varying health status (e.g., patients suffering from cancer, hemodialysis, traumatic brain injuries, eating disorders, and mechanically ventilated patients). In addition, critically ill patients reduced their RMR daily variability later during their hospital course and stabilization [24]. However, daily RMR variability does not inherently correlate with lower or higher resting EE values during the assessment (i.e., lower or higher kilocalories per day), and hence, all daily variability cannot be completely explained by confounding factors such as nursing care procedures or surgery [24]. Therefore, it is plausible that the individuals’ intra-assessment variability could be influenced by their gender [14], age [21,25], and health status [16,17,18,19,20,21,22,23]. Unfortunately, whether a relationship exists between the intra-assessment variability (i.e., intra-assessment CV for VO_2_, VCO_2_, RER, and resting EE) and health-related markers (e.g., body composition, circulating cardiometabolic risk factors, and cardiac autonomic function) has not been deeply explored. Considering all of these factors together, we hypothesized that men will present a higher intra-assessment RMR variability compared to women and that this intra-assessment RMR variability will be associated with classical health-related factors.

Thus, in the present exploratory study, we aimed to examine: (i) the intra-assessment RMR variability (expressed as CV (in percentage) for VO_2_, VCO_2_, RER, and resting EE) exhibited by men and women, separately; and (ii) whether the intra-assessment RMR variability (expressed as CV for VO_2_, VCO_2_, RER, and resting EE) is associated with health-related factors such as body composition, cardiorespiratory fitness (i.e., peak VO_2_ uptake), circulating cardiometabolic risk factors, heart rate (HR), and heart rate variability (HRV) parameters in a cohort of relatively healthy young adults. To the best of the authors’ knowledge, our present work is the first study exploring the relationship between the intra-assessment RMR variability and objectively determined health-related markers.

## 2. Materials and Methods

### 2.1. Study Subjects

The present cross-sectional study used pre-intervention (i.e., baseline) data from the ACTIBATE randomized control trial (RCT; ClinicalTrials.gov ID: NCT02365129) study [26,27]. A total of 107 relatively healthy young adults were included in the present study. All subjects provided both oral and written informed consent (see the *Institutional Review Board Statement* Section presented below for extended information). In brief, the inclusion criteria were: (i) being sedentary; (ii) maintaining a stable body weight (change lower than 3 kg over the last months); (iii) not being on a weight loss program; (iv) presenting a normal electrocardiogram; (v) not suffering from chronic (or acute) illness; (vi) not being a smoker; and (vii) not being pregnant or lactating. Extended and detailed information concerning the ACTIBATE study can be found elsewhere [26,27].

### 2.2. Resting Indirect Calorimetry Assessments

The VO_2_ and VCO_2_ gas exchanges were measured using either a CPX Ultima CardiO2 or a CCM Express metabolic cart (Medical Graphics Corp., St. Paul, MN, USA) during a 30-min period, while subjects were at rest (i.e., laying on bed in the supine position), in the morning (~9 AM), and following a 12 h overnight fast. Subjects were instructed not to perform moderate (24 h) and/or vigorous (48 h) exercise or physical activity before the assessment. Subjects were instructed to come to the lab by public transportation or motorized vehicle to avoid physical activity after they woke up. In addition, an acclimation period of 20–30 min was performed before the RMR assessment, as recommended by current guidelines [15]. During the entire gas exchange measurement, subjects were instructed to stay awake, remain silent, breathe normally, and avoid fidgeting [15].

Both metabolic carts mentioned above require a neoprene face mask equipped with a Directconnect™ low-flow sensor (Medical Graphics Corp., St. Paul, MN, USA), measured the VO_2_ and VCO_2_ using the same galvanic fuel cell and non-dispersive infrared analyzers [28], and require exactly the same calibration procedures. Before each RMR assessment, flow (using a 3 L syringe) and gas analyzers (using 2 gas bottles of standard gas concentrations) were calibrated accordingly to the manufacturers’ recommendations and instructions.

**Figure 1:**
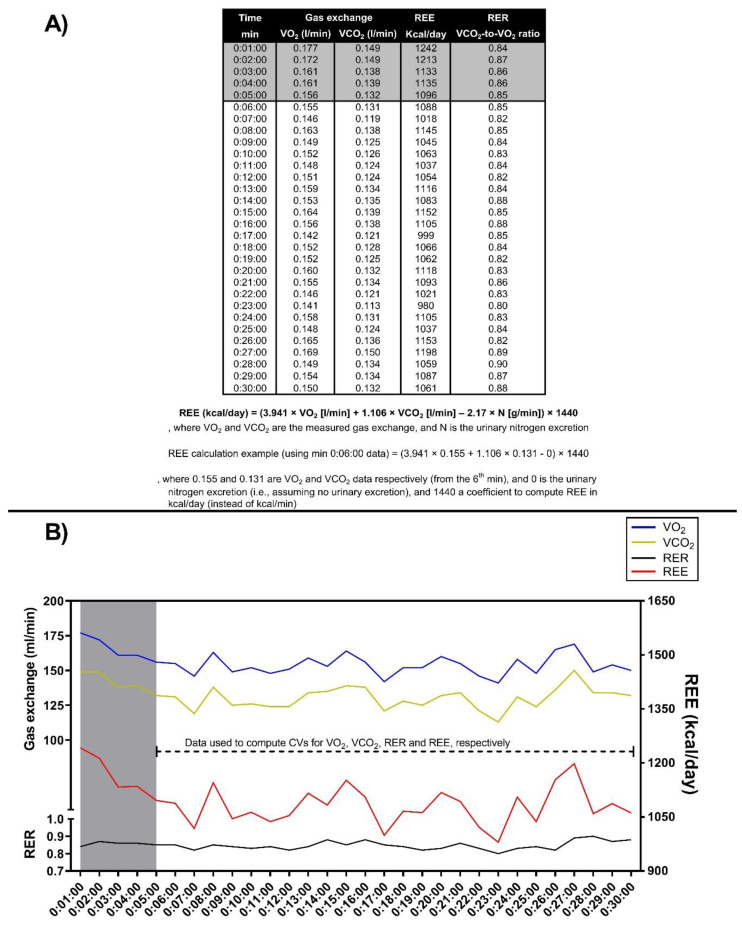
Representation of the procedure for computing the respiratory exchange ratio (RER) and the resting metabolic rate (RMR; i.e., resting energy expenditure [REE] estimated using the equation proposed by Weir [30] and assuming no urinary nitrogen excretion) using the measured volume of oxygen consumption (VO_2_) and volume of carbon dioxide production (VCO_2_) gas exchange (Panel (**A**)), and the procedure for computing each coefficient of variation (CV, in percentage; Panel B). These are actual data from a participant included in the present study. The blue line represents the VO_2_, the yellow line represents the VCO_2_, the black line represents the RER, and the red line represents the RMR (i.e., REE). In both panels, the data highlighted in gray represents the 5 min data discarded following current recommendations [15]. In Panel (**B**), the dashed line represents the data used to compute the CV for each gas exchange parameter (i.e., CV for VO_2_, VCO_2_, RER, and REE). In Panel B, the left *y*-axis represents the gas exchange in milliliters per minute (mL/min) and RER, and the right *y*-axis represents the REE in kilocalories per day (kcal/day).

After each RMR assessment, the resulting gas exchange data was downloaded using the metabolic carts’ specific software (MGCDiagnostic^®^ Breeze Suite, v. 8.1.0.54 SP7; Medical Graphics Corp., St. Paul, MN, USA) at a sampling frequency of 1 min, as extensively detailed elsewhere [29]. As recommended by current guidelines, the first 5 min of data were discarded [15], and thus, the remaining 25 min of data were processed and used for further calculations detailed below. The RMR (i.e., resting energy expenditure [REE]) was estimated using the equation proposed by Weir [30] and assuming no urinary nitrogen excretion (see Figure 1A). Then, the RER was calculated as the VCO_2_-to-VO_2_ ratio (see Figure 1A). In addition, for descriptive purposes, we computed the RMR relative to body weight (i.e., RMR/kg of body weight; RMR_BW_) and the RMR relative to fat-free mass (RMR/kg of fat-free mass; RMR_FFM_). Finally, as surrogate parameters of intra-assessment RMR variability, the CV for VO_2_, VCO_2_, RER, and RMR (thereinafter *CV for REE*) was calculated and expressed as a percentage for each subject (see Figure 1B). As an example, the CV for VO_2_ was computed for each participant as: (VO_2_ standard deviation/VO_2_ average) × 100. A summary of this entire process is depicted in Figure 1.

### 2.3. Anthropometry and Body Composition Assessments

The subject’s body weight and height were determined using a scale and a stadiometer (SECA model 799, Hamburg, Germany), respectively. Then, we computed BMI as body weight (kg)/height (m) squared. In addition, waist circumference was assessed twice, using a plastic tape, and the mean of both assessments was used for analyses. In this regard, we calculated a Z-score for waist circumference and used it to compute two different cardiometabolic risk Z-scores, which are detailed below.

Finally, body composition (fat mass, lean mass, and fat mass percentage) was assessed by a whole-body Dual Energy X-ray Absorptiometry scanner (Discovery Wi, Hologic Inc., Bedford, MA, USA).

### 2.4. Cardiorespiratory Fitness Assessment

The peak VO_2_ uptake (i.e., the cardiorespiratory fitness; CRF) was determined by indirect calorimetry (CPX Ultima CardiO2, Medical Graphics Corp., St. Paul, MN, USA; metabolic cart information was detailed above), while subjects elicited a maximum-effort graded exercise protocol [31]. As for the resting assessments, we calibrated both the volume (using a 3 L syringe and a high-flow sensor [Medical Graphics Corp., St. Paul, MN, USA]) and gas analyzers (using 2 gas bottles of standard gas concentrations) prior to every test, following the manufacturers’ recommendations. The CPX Ultima CardiO2 is a breath-by-breath metabolic cart that equipped a galvanic fuel cell for measuring VO_2_ and a non-dispersive infrared analyzer for measuring VCO_2_ (resolution of both gas analyzers ± 0.1%). Regarding the exercise protocol, briefly, every 1-min, the slope of the treadmill (H/P/cosmos pulsar; H/P/cosmos sports & medical GmbH, Nussdorf-Traunstein, Germany) increased by 1%, while subjects walked at 5.3 km/h until volitional exhaustion (self-reported by the subject). In addition, during the entire exercise protocol, the heart rate was monitored using an electrocardiogram. We considered that subjects performed their maximum effort when they met the following criteria [32]: (i) the RER was ≥1.1; (ii) the self-reported perceived exertion was >9 using the rating of perceived exertion category-ratio scale (RPE-CR10) [33]; and (iii) the heart rate was ≥90% of the age-predicted maximum heart rate (i.e., 209 − 0.73 × age). Lastly, all subjects were instructed not to consume any stimulant substance (e.g., caffeine) before the test (24 h), not to eat (3–5 h), and perform neither intense/vigorous nor moderate physical activity during the previous 48 h and/or the previous 24 h, respectively. Extended information can be found elsewhere [34].

To compute the peak VO_2_ uptake, we first downloaded the data at a sample frequency of 5 s. Subsequently, from the entire set of recorded data, we looked for the highest VO_2_ uptake value and then averaged the peak (i.e., highest) VO_2_ uptake value and the immediately 5-s values prior and after the peak VO_2_ uptake value (i.e., we used a 15-s data average to be used as that peak VO_2_ uptake value) [34]. Of note, as an approach to detect possible artefacts in the assessment (e.g., artefacts that may unmask the real VO_2_ value), we checked the entire data set from the 2nd to the 10th subsequent highest VO_2_ uptake values (i.e., the 2nd peak VO_2_ uptake value, the 3rd, the 4th, etc.) [34]. In addition, we computed the CRF relative to body weight (i.e., peak VO_2_ uptake/kg of body weight; CRF_BW_) and the CRF relative to fat-free mass (peak VO_2_ uptake/kg of fat-free mass; CRF_FFM_).

### 2.5. Circulating Cardiometabolic Risk Factors and Blood Pressure Assessments

For determining circulating cardiometabolic risk factors, subjects rested (sat) for at least 10 min and underwent an overnight fast of at least 12 h. Then, blood samples were collected from the antecubital vein in Vacutainer^®^ SST™ II Advance tubes (Becton Dickinson, Plymouth, UK) to obtain serum. After collection, tubes were centrifuged, aliquoted, and stored (−80 °C) for later analyses.

Total cholesterol, high- and low-density lipoprotein cholesterol (HDL-C and LDL-C, respectively), triglycerides, and glucose were determined by spectrophotometry (model AU5800; Beckman Coulter, Brea, CA, USA), while insulin was determined by chemiluminescence immunoassay involving UniCel DxI 800 paramagnetic particles (Beckman Coulter, Brea, CA, USA). Finally, using the determined blood glucose and insulin values, the homeostatic model assessment of insulin resistance index (HOMA index) was computed:

$$\mathrm{HOMA}\;\mathrm{index}=\frac{\mathrm{Insulin}\;\times \;\mathrm{Glucose}}{\begin{array}{c} 22.5 \end{array}}$$

An automatic monitor (HEM 705 CP; Omron Healthcare Co., Kyoto, Japan) was used to assess blood pressure (BP). Two BP measurements were performed (in the right arm) while subjects were resting, and mean values were used for further analyses.

Finally, we computed a cardiometabolic risk Z-score (hereinafter cardiometabolic risk Z-score 1) using classical metabolic syndrome markers, i.e., glucose, triglycerides, HDL-C, BP, and waist circumference. To compute that cardiometabolic risk Z-score, we computed the individual Z-score for each of these markers as: (value − mean)/standard deviation. Regarding the Z-score for the HDL-C, only for this marker were its values inverted (i.e., multiplied by −1); thus, higher values in the Z-score can be interpreted as higher cardiometabolic risk. After computing all individual Z-scores, the cardiometabolic risk Z-score (i.e., the Z-score including all the individual Z-scores) was computed as detailed below:

$$\begin{array}{c} \mathrm{Cardiometabolic}\;\mathrm{risk}\;Z-\mathrm{score}\;1\\ =\frac{\left(Z-\mathrm{score}\;\mathrm{glucose}\;+\;Z-\mathrm{score}\;\mathrm{triglycerides}\;+\;Z-\mathrm{score}\;\mathrm{HDL}\;-\;C\;+\;Z-\mathrm{score}\;\mathrm{mean}\;\mathrm{blood}\;\mathrm{pressure}\;+\;Z-\mathrm{score}\;\mathrm{waist}\;\mathrm{circumference}\right)}{\begin{array}{c} 5 \end{array}} \end{array}$$

Moreover, we computed a second cardiometabolic risk Z-score (hereinafter cardiometabolic risk Z-score 2), adding to the metabolic syndrome markers mentioned above in the cardiometabolic risk Z-score 1 the TC, the LDL-C, the insulin, and the HOMA index Z-score values.

### 2.6. Heart Rate and Heart Rate Variability Assessment

The heart rhythm was recorded over a 15-min period using a Polar RS800CX (Polar Electro, Kempele, Finland; sample frequency 1000 Hz), early in the morning (between 8 and 9 AM), while the subjects were lying on a bed (awake, in the supine position). Of note, this measurement was performed immediately before the indirect calorimetry assessment (i.e., in the same room and under the same ambient temperature and humidity conditions). In addition, subjects were instructed to stay awake, not move too much (i.e., be motionless), and remain silent while the heart rhythm was recorded.

The heart rhythm data was processed using the Kubios software (free version, v.3.0.0, HRV analysis, University of Eastern Finland) [35]. In brief, we excluded the first 5 min of data and manually selected the 5-min period [36,37]. Subsequently, in the selected 5-min period, we applied the medium Kubios threshold-based artefact correction level following current recommendations [38]. Finally, the R-R interval series were detrended using the smoothness prior method with alpha set at 500 ms and a cubic interpolation at the default rate of 4 Hz [36,38].

From the heart rhythm recorded, we derived the HR (in beats per minute). In addition, and following the Guidelines of The European Society of Cardiology task force and The North American Society of Pacing and Electrophysiology [39], we derived the vagal-related HRV variables from time and frequency domains. In the first domain, we derived: (i) the squared root of the mean of the sum of the squares of successive R-R interval differences (RMSSD); (ii) the standard deviation of all normal R-R intervals (SDNN); and (iii) the percentage of pairs of adjacent R-R intervals differing by more than 50 milliseconds (pNN50). In the frequency-domain, we derived the power of the high frequency band (HF; 0.15–0.4 Hz) using the Fast Fourier transformation algorithm.

Finally, we also computed the vagal-related HRV score as proposed in our previous study [40]. In brief, we calculated an individual Z-score for all the aforementioned time and frequency domain parameters (i.e., the RMSSD, the SDNN, the pNN50, and the HF), and subsequently, we ran the following equation [40]:

$$\mathrm{HRV}\;\mathrm{score}=\frac{\left(Z-\mathrm{score}\;\mathrm{RMSSD}\;+\;Z-\mathrm{score}\;\mathrm{SDNN}\;+\;Z-\mathrm{score}\;\mathrm{pNN}50+\;Z-\mathrm{score}\;\mathrm{HF}\right)}{\begin{array}{c} 4 \end{array}}$$

### 2.7. Statistical Analyses

The normal distribution of the variables was examined using the Kolmogorov-Smirnov test and the visual inspection of histograms. As part of our descriptive analyses, for all health-related outcomes and for both RMR ratios, we compared men vs. women using non-paired t-tests. Then, for analytical purposes, the variables that presented a skewed distribution were transformed using a natural logarithm (ln). Analyses were performed separately for men and women as suggested by previous literature (e.g., [14]). Although the sample sizes were different (n = 35 and n = 72 for men and women, respectively), we performed an analysis of covariance (ANCOVA) with Bonferroni comparisons to examine sex-adjusted mean differences (age as a confounder factor) on indirect calorimetry parameters (CVs for VO_2_, VCO_2_, RER, and REE). Multiple linear regressions analyses, adjusting for age, were conducted to test the associations between the CVs obtained for the assessed indirect calorimetry parameters (i.e., the CVs for VO_2_, VCO_2_, RER, and REE) and: (i) anthropometric and body composition parameters; (ii) CRF (expressed as absolute and relative values); (iii) circulating cardiometabolic risk factors and BP; and (iv) HR and HRV derived parameters. In addition, the associations between the abovementioned CVs and both cardiometabolic risk Z-scores (i.e., cardiometabolic risk Z-scores 1 and 2) and the vagal-related HRV score, adjusting for age, were also conducted.

Analyses were performed using the Statistical Package for the Social Sciences v.22.0 (IBM SPSS Statistics, IBM Corporation, Chicago, IL, USA). The significance level was set at *p* < 0.05. The results are presented as mean ± standard deviation (SD), and as standardized β unless otherwise stated. Graphs were created using the software Graph Pad Prism (GraphPad, v. 8.0.2, San Diego, CA, USA).

## 3. Results

A total of 35 men (age = 23 ± 2 years old) and 72 women (age = 22 ± 2 years old) were included in the study. Table 1 provides descriptive data for the participants in both groups (i.e., men and women). In brief, mean differences were observed in all anthropometry and body composition parameters except fat mass (expressed in kilograms; Table 1). In addition, mean CRF and CR_FBW_ were higher in men compared to women (Table 1). Concerning circulating cardiometabolic risk factors and blood pressure parameters, men showed lower mean HDL-C, higher mean systolic BP, and higher cardiometabolic risk Z-score values (Table 1). Finally, no mean differences were observed for HR and HRV-related outcomes (Table 1). Regarding RMR ratios, we observed no mean differences in RMR_BW_ between men and women (21.2 ± 4.5 kcal/kg/day vs. 22.1 ± 3.4 kcal/kg/day; *p* = 0.299), but mean differences were observed for the RMR_FFM_ ratio (30.1 ± 5.0 kcal/kg/day vs. 36.4 ± 4.7 kcal/kg/day for men and women, respectively; *p* < 0.001).

**Figure 2:**
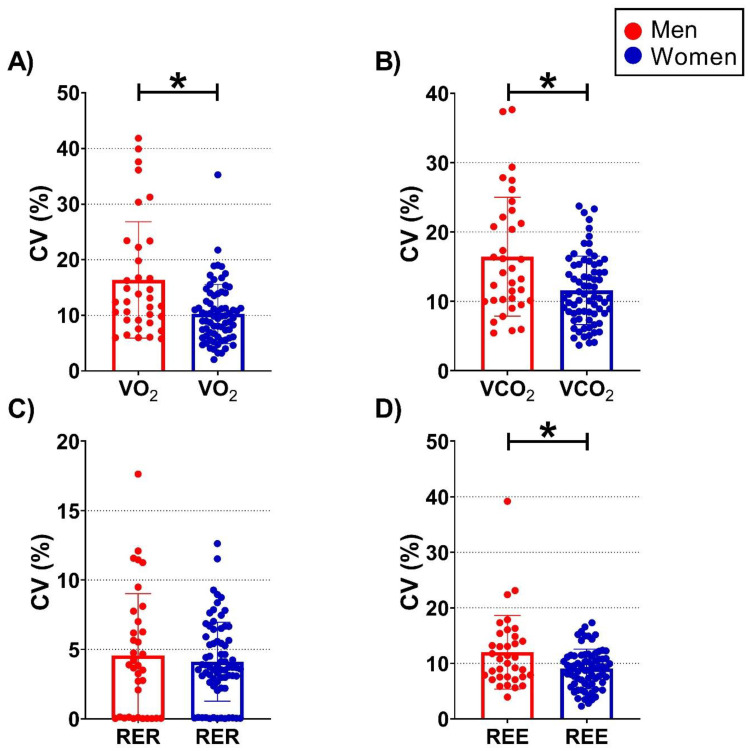
Column plots for each coefficient of variation (CV, expressed as a percentage) of the data separated by men (red circles) and women (blue circles). Results are presented as mean and standard deviation, and all data points (i.e., individual values). CV were calculated for each individual and parameter, i.e., for the volume of oxygen consumption (VO_2_; Panel (**A**)), for the volume of carbon dioxide production (VCO_2_; Panel (**B**)), for the respiratory exchange ratio (RER; Panel (**C**)), and for the resting metabolic rate (RMR; i.e., resting energy expenditure [REE] estimated using the equation proposed by Weir [30] and assuming no urinary nitrogen excretion; Panel (**D**)). * represents *p*-values < 0.05 derived from analysis of covariance (ANCOVA) to examine between sex (i.e., men vs. women) adjusted mean differences while taking participants’ age into account as a confounder (i.e., ANCOVA models adjusted for age). N = 35 and 72 for men and women, respectively.

The intra-assessment variability, the individual CVs for VO_2_, VCO_2_, RER, and REE for men and women, are depicted in Figure 2. In general terms, men had a higher intra-assessment variability for all parameters. Men presented higher CVs for VO_2_ (adjusted mean difference of 5.9 ± 1.5%; *p* < 0.001; Figure 2A), for VCO_2_ (adjusted mean difference of 4.8 ± 1.3%; *p* < 0.001; Figure 2B), and for REE (adjusted mean difference of 3.4 ± 1.0%; *p* = 0.001; Figure 2D), compared to women. No differences were observed for CV for RER (adjusted mean difference of 0.59 ± 0.71%; *p* = 0.409; Figure 2C). Of note, some men presented CVs for VO_2_, VCO_2_, and REE (only one participant for this parameter) > 35% (Figure 2, red circles).

Table 2 shows the results from multiple linear regression analyses between the CVs obtained for the indirect calorimetry parameters and the anthropometric and body composition parameters, the CRF (expressed as absolute and relative values), circulating cardiometabolic risk factors and BP, HR and HRV derived parameters, both cardiometabolic risk Z-scores, and the vagal-related HRV score (all associations were performed separately for men and women and adjusted for age). For men, we observed a negative association between the CV for RER and vagal-related HRV parameters, specifically the RMSSD and the pNN50 (β = −0.36 and −0.38, respectively; both *p* < 0.04; Table 2). Moreover, a positive association was observed between the CV for RER and the mean HR (β = 0.41; *p* < 0.03) for men. Concerning the women group, we observed no associations between the CVs for VO_2_, VCO_2_, RER, and REE and the health-related parameters (all *p* > 0.05; Table 2).

## 4. Discussion

This study represents a first exploration of the intra-assessment RMR variability (expressed as CV for VO_2_, VCO_2_, RER, and REE) exhibited by young and relatively healthy men and women and its relationship with health-related parameters. Our results show a remarkable intra-assessment RMR variability, which was significantly greater in men than in women (Figure 2A,B,D). We also found that in men, the intra-assessment RMR variability for the assessed RER variability (i.e., CV for RER) was negatively associated with RMSSD and pNN50 and positively associated with mean HR (Table 2). However, in women, no associations were observed between the intra-assessment RMR variability and the different parameters included in the study.

Previous literature showed that certain anthropometric characteristics such as body weight and height and body composition (e.g., fat-free mass) are directly related to energy expenditure and RMR [41,42,43]. We should highlight that our groups were different in all anthropometric and body composition parameters, except fat mass. In brief, men were heavier, taller, and presented a higher FFM compared to women (Table 1). However, these anthropometric differences may be explained by the fact that men presented an obese phenotype (based on BMI and waist circumference values; Table 1). After accounting for “body size”, the mean RMR of men was similar to that yielded by women, as suggested by the RMR_BW_ ratio (*p* = 0.299). However, after considering the FFM (i.e., RMR_FFM_), we observed that men expended less energy during resting compared to women 30.1 ± 5.0 vs. 36.4 ± 4.7 kcal/kg/day (*p* < 0.001). Interestingly, this issue should be acknowledged, as low RMR values could predispose to weight (re)gain [44], since the RMR component could account for up to 70% of the 24-h energy expenditure of sedentary individuals [7]. On the other hand, and regarding variation in EE components, a recent study by Halsey et al. [14] observed that after comparing men and women of similar characteristics (i.e., same age, height, and fat and fat-free masses), men presented a larger variation in different EE components, including total EE, activity EE (the authors estimated this component as: 0.9 × total EE − basal EE), and basal EE. Thus, based on their results, it is reasonable to hypothesize that morphometric and body composition parameters would have an impact on the intra-assessment RMR variability (i.e., CV for VO_2_, VCO_2_, RER, and REE). However, we did not observe a relationship between intra-assessment RMR variability and morphometric and body composition parameters (Table 2). Notably, this lack of association was consistent in both men and women.

Concerning CRF, no association was observed between the intra-assessment RMR variability and the CRF, regardless of whether it was expressed as absolute or relative (CRF_BW_ or CRF_FFM_) values (Table 2). As occurred previously for the anthropometric parameters, the same results were observed for men and women. This absence of association could be explained, at least partially, by the fact that our sample was composed of sedentary individuals, thus this issue may have influenced our results to an unknown extent, as regular exercise and/or physical activity directly influence several systems and organs [45]. Halsey et al. [14] suggested that the dimorphism (i.e., greater basal energy expenditure variability) observed in men compared to women could be influenced by adaptations produced by exercise and training. Congruently, it is known that physical activity levels are more variable in men than in women [46], and thus Halsey et al. [14] proposed that these adaptations may partially drive the observed differences in variability between men and women.

Contrary to our expectations, no associations were observed between the intra-assessment RMR variability and all the circulating cardiometabolic risk factors included in our study. In this case, the absence of associations was consistent in men and women, as were the systolic and diastolic BP assessments (Table 2). This could be partially explained by the fact that participants were young and metabolically healthy according to their body composition and cardiometabolic risk factor levels (Table 1). In fact, these results concur with previous literature in which an exercise intervention carried out in relatively healthy individuals did not induce changes in the assessed cardiometabolic risk factors [47,48], as well as with the results observed in the ACTIBATE study [27], an RCT from which the data of the present study arose. Trying to overcome this possible limitation, we computed two different cardiometabolic risk Z-scores (see Materials and Methods Section) to further study the association between intra-assessment RMR variability and circulating cardiometabolic risk factors. However, the results remained unaltered even after computing both cardiometabolic risk Z-scores (Table 2).

Finally, we observed that certain vagal-related HRV-derived parameters, which are widely employed as surrogate markers of cardiometabolic health [40], were inversely associated with CV for RER in men (Table 2). In a previous study, we also observed that HRV was differently associated with circulating cardiometabolic risk factors in men and women [49]. In brief, we computed three different HRV ratios and studied whether they were similarly associated with circulating cardiometabolic risk factors in young men and women [49]. Interestingly, we observed that the HRV ratios were associated with the circulating cardiometabolic risk factors in the group of young women but not in men. Here, we observed a positive association between the intra-assessment RER variability and HR and a negative association with vagal-related HRV parameters such as RMSSD and pNN50 in men (Table 2). Previous literature showed that, while the subjects are in the resting state (e.g., sitting or lying), those individuals exhibiting lower values of vagal-related HRV parameters (e.g., low RMSSD values) present a higher risk of suffering from cardiovascular diseases and an increased morality risk [39,50,51,52]. Moreover, literature have also suggested that these individuals presenting higher HR values in the resting state present a worse health status compared to these individuals presenting lower HR values [34,40,53,54]. This is in line with a recent meta-analysis of prospective studies, in which Aune et al. [55] observed an increased risk of suffering from cardiovascular diseases and all-cause mortality in those subjects exhibiting greater resting HR values. Therefore, our results show that men exhibiting a higher CV for RER presented a higher cardiometabolic risk (as suggested by the directions of the associations mentioned above; Table 2). To the best of our knowledge, this is the first study investigating whether the intra-measurement RMR variability is related to HR and HRV in young men and women, and therefore, we cannot perform comparisons between studies. Nevertheless, this relationship is of interest as it could be related to metabolic flexibility, a parameter of interest due to its possible relationship with cardiometabolic health [56]. In brief, metabolic flexibility is considered the ability to shift from one substrate (e.g., carbohydrates) to another (e.g., fat) based on fuel availability [56], an ability that is widely considered a cardiometabolic health marker. In this regard, during prolonged fasting, the endogenous fat concentration increases, stimulating fat oxidation [57]. Based on the fasting state of our participants (12 h), they should theoretically be predominantly oxidizing fat, and thus, the expected CV for RER should be low. However, we observed a high CV for RER in men (Figure 2), which may indicate that they were alternating from one substrate to another, thereby suggesting a putatively impaired metabolic flexibility in comparison to women. This issue suggests that exhibiting a high CV for RER in resting conditions could be a potential marker of cardiometabolic risk. Of note, about 90% of VO_2_ is coupled to adenosine triphosphate production by the mitochondria, with nearly 19−28% being used by the sodium–potassium pump [3], and approximately 20−30% being coupled to the basal mitochondrial proton and electron leak [58]. Thus, variation in RMR between individuals, populations, and/or species could be mostly attributed to differences in mitochondrial function [59], although individual differences in humans are mostly explained by a different mitochondrial O_2_ affinity [60]. Unfortunately, our study design precludes further study of this issue and whether differences in mitochondrial O_2_ affinity might be mediating our observations. Future studies involving different study populations (e.g., unhealthy vs. healthy participants), study designs (e.g., muscle biopsies and mitochondrial respiration analyses), and larger cohorts are needed to confirm our results.

Growing evidence also highlights the potential effect of the subjects’ dietary habits on the RMR and the energy balance of the individuals [61,62,63]. In fact, diet seems not only to modulate gut microbiota composition but also energy balance [63,64]. In line with this, a recent study that compared the effects of the Mediterranean diet—the dietary pattern that likely resembles the diet followed by our study participants—vs. a vegan diet found that the Mediterranean diet positively shaped salivary microbiota composition (higher abundance of *Subflava* and *Prevotella* species) [61]. Specifically, *Prevotella* abundance was inversely associated with RER, whereas *Subflavan* abundance was positively associated with RMR [61]. Unfortunately, in their study, the authors did not analyze whether salivary microbiota were further associated with any of the intra-assessment RMR variability parameters of our study [61]. Considering all these findings, future studies evaluating the association between intra-assessment RMR variability and health-related factors should also consider not only the impact of the diet followed by the individuals but also the individuals’ gut and/or salivary microbiota composition as possible modulators of human metabolism and energy balance.

Considering all of this together, and although no associations were observed between the intra-assessment RMR variability and certain health-related factors included in our present study (only associations with HR, RMSSD, and pNN50 were observed), we recommend its inclusion in future studies. In this regard, computing the CV for the gas exchange outcomes is an easy and feasible procedure that does not require additional measurements and, furthermore, can be retrospectively calculated. Thus, considering our results and these advantages, the intra-assessment RMR variability expressed as CV should be considered in the future as a potential marker for evaluating cardiometabolic health. There were a few limitations to our study that deserve attention. The assessment of RMR (thus, intra-assessment RMR variability) was performed using two different metabolic carts equipped with a face mask. Therefore, the use of other metabolic carts or other gas exchange collection systems (e.g., a canopy hood system) may influence the results. The cross-sectional design of our study does not allow us to establish any cause-and-effect relationships, thus, longitudinal studies in which the intra-assessment RMR variability as well as the health-related parameters are measured at different time points within the same participant are needed. Furthermore, it is important to consider that our study was performed in young and relatively healthy adults; thus, studies carried out in other populations (e.g., older populations, ill patients, etc.) and in larger cohorts to increase the statistical power of the analyses are warranted. Finally, we assessed the heart rhythm using a heart rate monitor instead of an electrocardiograph. However, we used the Polar RS800CX heart rate monitor, which has been validated against the gold-standard technique (i.e., electrocardiography); in this regard, the heart rate monitor used in our study is considered a valid instrument for the heart rhythm recording [65,66].

## 5. Conclusions

Young and relatively healthy men exhibit a remarkable intra-assessment RMR variability in terms of CV for VO_2_, VCO_2_, and REE that is greater than that observed for women. Conversely, no differences in CV for RER were observed between men and women. Moreover, in men, the intra-assessment RER variability was negatively associated with RMSSD and pNN50 and positively associated with mean HR. Interestingly, in women, no associations were observed between the intra-assessment RMR variability and the diverse health-related parameters included. Our results suggest that intra-assessment RMR variability, and specifically the CV for RER, could be a potential marker of cardiometabolic risk in young and relatively healthy men. The present study provides novel, preliminary findings suggesting a sexual dimorphism in the association between intra-assessment RMR variability and health-related factors in young, relatively healthy adults.

## Figures and Tables

**Table 1:** Participants’ descriptive characteristics.

	Men (n = 35)		Women (n = 72)		
	Mean	SD	Mean	SD	*p*
Anthropometry and body composition parameters					
Weight (kg)	82	16	63	12	<0.001
Height (cm)	175	7	164	7	<0.001
BMI (kg/m^2^)	27	5	23	4	<0.001
Fat mass (kg)	25	11	24	8	0.558
Fat mass (%)	30	7	38	6	<0.001
Fat free mass (kg)	55	7	38	5	<0.001
Waist circumference (cm)	89	14	76	11	<0.001
Cardiorespiratory fitness parameters					
CRF (ml/min)	3745	710	2528	433	<0.001
CRF_BW_ (ml/[kg/BW]/min)	46.2	8.9	40.7	5.9	0.002
CRF_FFM_ (ml/[kg/FFM]/min)	67.7	9.5	67.0	8.1	0.708
Circulating cardiometabolic risk factors and blood pressure parameters					
Glucose (mg/dl)	89	7	86	5	0.052
Insulin (UI/ml)	9	6	7	3	0.257
HOMA index	2	2	2	1	0.185
Total cholesterol (mg/dl)	161	32	167	32	0.431
HDL-C (mg/dl)	45	8	55	11	<0.001
LDL-C (mg/dl)	98	28	96	24	0.707
Triglycerides (mg/dl)	88	45	77	42	0.256
Systolic BP (mm Hg)	127	12	112	11	<0.001
Diastolic BP (mm Hg)	70	12	67	8	0.122
Cardiometabolic risk Z-score 1	0.60	0.70	−0.30	0.50	<0.001
Cardiometabolic risk Z-score 2	0.40	0.80	−0.20	0.50	0.002
Heart rate and heart rate variability parameters					
Mean HR (bpm)	67	11	69	9	0.537
RMSSD (ms)	59	34	62	32	0.694
SDNN (ms)	54	25	53	23	0.888
pNN50 (%)	33	23	36	21	0.513
HF (ms^2^)	7	1	7	1	0.380
HRV Z-score	0.01	1.00	0.10	1.00	0.700

Results are presented as mean and standard deviation (SD), and *p*-values from students t-test analyses. BMI: body mass index; CRF: cardiorespiratory fitness (i.e., peak VO_2_ uptake); CRF_BW_: CRF relative to body weight (peak VO_2_ uptake/kg of body weight); CRF_FFM_: CRF relative to fat-free mass (peak VO_2_ uptake/kg of fat-free mass); HOMA: homeostatic model assessment of insulin resistance index; HDL-C, high-density lipoprotein cholesterol; LDL-C, low-density lipoprotein cholesterol; BP, blood pressure; Cardiometabolic risk Z-scores 1 and 2: the first Z-score included classical metabolic syndrome markers (glucose, triglycerides, HDL-C, BP, and waist circumference), while the second included these classical metabolic syndrome markers and insulin related markers (insulin and HOMA index) and total cholesterol and LDL-C Z-score values; HR, heart rate (in beats per minute [bpm]); RMSSD, the square root of the mean of the sum of the squares of the R-R interval differences; SDNN, standard deviation of normal R-R intervals; pNN50, the percentage of R-R intervals that show a difference higher than 50 milliseconds (ms); HF, the power of the high-frequency band (HF: 0.15–0.4 Hertz); HRV Z-score: heart rate variability Z-score including the RMSSD, the SDNN, the pNN50, and the HF time and frequency domain parameters’ Z-score values.

**Table 2:** Association between the intra-assessment resting metabolic rate (RMR) variability expressed as a coefficient of variation (CV) for each of the assessed indirect calorimetry parameters, i.e., the CVs for volume of oxygen consumption (VO_2_), volume of carbon dioxide production (VCO_2_), respiratory exchange ratio (RER), and resting energy expenditure (REE), and different health-related parameters.

	CV for VO_2_				CV for VCO_2_				CV for RER				CV for REE			
	Men		Women		Men		Women		Men		Women		Men		Women	
	β	*p*	β	*p*	β	*p*	β	*p*	β	*p*	β	*p*	β	*p*	β	*p*
Anthropometry and body composition parameters																
Weight (kg)	−0.100	0.558	0.103	0.851	−0.172	0.308	0.030	0.800	−0.158	0.351	−0.066	0.598	−0.154	0.362	−0.025	0.838
Height (cm)	0.070	0.695	0.067	0.580	0.067	0.709	−0.043	0.716	0.079	0.656	−0.123	0.317	0.134	0.453	−0.045	0.709
BMI (kg/m^2^)	−0.143	0.393	0.054	0.661	−0.215	0.193	0.040	0.740	−0.195	0.240	−0.009	0.942	−0.214	0.197	−0.013	0.916
Fat mass (kg)	−0.190	0.272	0.117	0.336	−0.244	0.155	0.064	0.594	−0.109	0.530	−0.065	0.603	−0.218	0.205	−0.022	0.855
Fat mass (%)	−0.210	0.231	0.081	0.508	−0.235	0.177	0.083	0.493	−0.061	0.729	−0.037	0.769	−0.228	0.192	−0.021	0.865
Fat free mass (kg)	0.093	0.585	0.040	0.745	0.010	0.953	−0.033	0.784	−0.177	0.291	−0.040	0.751	0.012	0.943	−0.033	0.783
Waist circumference (cm)	−0.086	0.613	0.126	0.301	−0.167	0.320	0.032	0.794	−0.257	0.122	−0.161	0.195	−0.256	0.125	−0.091	0.453
Cardiorespiratory fitness parameters																
CRF (mL/min)	0.042	0.821	−0.017	0.894	−0.054	0.769	−0.048	0.698	−0.283	0.116	−0.006	0.964	−0.104	0.576	−0.060	0.623
CRF_BW_ (mL/[kg/BW]/min)	0.082	0.650	−0.135	0.273	0.068	0.706	−0.085	0.489	−0.112	0.530	0.084	0.507	0.063	0.728	−0.038	0.758
CRF_FFM_ (mL/[kg/FFM]/min)	0.005	0.978	−0.075	0.543	−0.038	0.835	−0.028	0.818	−0.177	0.325	0.049	0.696	−0.019	0.918	−0.048	0.693
Circulating cardiometabolic risk factors and blood pressure parameters																
Glucose (mg/dL)	−0.158	0.390	0.071	0.560	−0.037	0.839	−0.014	0.907	0.260	0.153	−0.072	0.564	−0.025	0.893	−0.044	0.713
Insulin (UI/mL)	−0.185	0.299	0.199	0.096	−0.253	0.150	0.098	0.408	−0.017	0.925	−0.020	0.872	−0.277	0.123	0.081	0.499
HOMA index	−0.162	0.367	0.218	0.072	−0.140	0.436	0.159	0.186	0.022	0.904	−0.020	0.876	−0.157	0.391	0.127	0.289
Total cholesterol (mg/dL)	0.099	0.589	0.016	0.896	0.149	0.412	0.115	0.330	−0.056	0.761	0.004	0.974	0.030	0.873	0.074	0.528
HDL-C (mg/dl)	0.206	0.253	−0.148	0.224	0.182	0.313	−0.158	0.187	−0.119	0.514	−0.082	0.509	0.108	0.559	−0.162	0.177
LDL-C (mg/dl)	0.086	0.638	−0.014	0.906	0.132	0.468	0.092	0.435	−0.065	0.723	0.009	0.939	0.021	0.912	0.065	0.584
Triglycerides (mg/dl)	−0.002	0.990	−0.057	0.640	−0.051	0.781	−0.067	0.575	0.091	0.623	−0.043	0.728	−0.083	0.659	−0.140	0.240
Systolic BP (mm Hg)	0.081	0.656	0.046	0.701	−0.013	0.944	0.069	0.562	−0.231	0.196	0.049	0.692	−0.046	0.800	0.073	0.542
Diastolic BP (mm Hg)	−0.023	0.898	0.043	0.726	−0.148	0.395	−0.090	0.454	−0.235	0.170	−0.133	0.286	−0.264	0.123	−0.114	0.345
Cardiometabolic risk Z-score 1	−0.097	0.600	0.066	0.588	−0.152	0.409	0.046	0.701	−0.090	0.628	0.010	0.934	−0.229	0.218	−0.046	0.702
Cardiometabolic risk Z-score 2	−0.062	0.740	0.062	0.614	−0.129	0.487	0.070	0.562	−0.096	0.606	0.009	0.940	−0.195	0.296	−0.015	0.900
Heart rate and heart rate variability parameters																
Mean HR (bpm)	−0.145	0.443	−0.039	0.771	−0.069	0.717	0.054	0.680	0.407	0.025	0.088	0.521	0.086	0.651	0.066	0.616
RMSSD (ms)	0.085	0.643	0.030	0.823	−0.015	0.933	−0.012	0.928	−0.364	0.039	−0.071	0.602	−0.040	0.828	−0.021	0.873
SDNN (ms)	0.070	0.702	−0.046	0.730	−0.023	0.900	−0.028	0.834	−0.330	0.062	0.071	0.606	−0.074	0.686	−0.028	0.833
pNN50 (%)	0.121	0.503	0.120	0.362	0.010	0.955	0.023	0.862	−0.376	0.031	−0.082	0.550	−0.047	0.795	0.036	0.787
HF (ms^2^)	0.074	0.683	0.018	0.891	−0.013	0.943	−0.076	0.563	−0.288	0.101	−0.074	0.586	−0.033	0.855	−0.025	0.848
HRV Z-score	0.047	0.800	−0.008	0.949	0.002	0.989	−0.044	0.737	−0.231	0.201	−0.070	0.611	−0.003	0.988	−0.026	0.844

Results are presented as standardized β and *p*-values from multiple linear regression analyses (adjusted for age). BMI: body mass index; CRF: cardiorespiratory fitness (i.e., peak VO_2_ uptake); CRF_BW_: CRF relative to body weight (peak VO_2_ uptake/kg of body weight); CRF_FFM_: CRF relative to fat free mass (peak VO_2_ uptake/kg of fat-free mass); HOMA: homeostatic model assessment of insulin resistance index; HDL-C, high-density lipoprotein cholesterol; LDL-C, low-density lipoprotein cholesterol; BP, blood pressure; Cardiometabolic risk Z-scores 1 and 2: the first Z-score included classical metabolic syndrome markers (glucose, triglycerides, HDL-C, BP, and waist circumference), while the second included these classical metabolic syndrome markers and insulin-related markers (insulin and HOMA index) and total cholesterol and LDL-C Z-score values; HR: heart rate (in beats per minute [bpm]); RMSSD: the square root of the mean of the sum of the squares of the R-R interval differences; SDNN: standard deviation of normal R-R intervals; pNN50: the percentage of R-R intervals that shows a difference higher than 50 milliseconds (ms); HF: the power of the high frequency band (HF: 0.15–0.4 Hertz); HRV Z-score: heart rate variability Z-score including the RMSSD, the SDNN, the pNN50, and the HF time and frequency domains parameters’ Z-score values.

## Data Availability

The data that support the findings of this study are available from the corresponding author, J.M.A.A., upon reasonable request. The data are not publicly available.
